# From Layer-by-Layer Growth to Nanoridge Formation: Selective Area Epitaxy of GaAs by MOVPE

**DOI:** 10.1021/acs.cgd.3c00316

**Published:** 2023-06-12

**Authors:** Nicholas Morgan, Vladimir G. Dubrovskii, Ann-Kristin Stief, Didem Dede, Marie Sanglé-Ferrière, Alok Rudra, Valerio Piazza, Anna Fontcuberta i Morral

**Affiliations:** † Laboratory of Semiconductor Materials, Institute of Materials, Ecole Polytechnique Fédérale de Lausanne, 1015 Lausanne, Switzerland; ‡ Faculty of Physics, Saint Petersburg State University, Universitetskaya Embankment 13B, 199034 St. Petersburg, Russia; § Laboratory of Semiconductor Materials, Institute of Physics, Ecole Polytechnique Fédérale de Lausanne, 1015 Lausanne, Switzerland

## Abstract

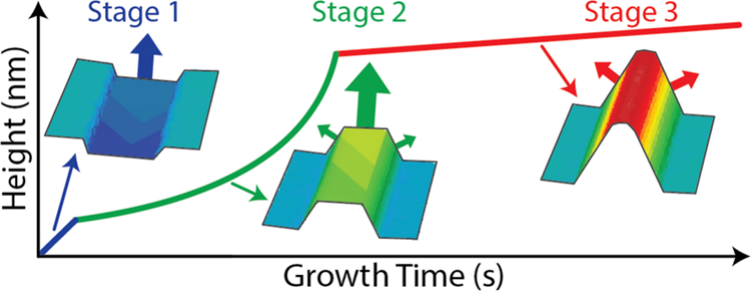

Selective area epitaxy at the nanoscale enables fabrication of high-quality nanostructures in regular arrays with predefined geometry. Here, we investigate the growth mechanisms of GaAs nanoridges on GaAs (100) substrates in selective area trenches by metal–organic vapor-phase epitaxy (MOVPE). It is found that pre-growth annealing results in the formation of valley-like structures of GaAs with atomic terraces inside the trenches. MOVPE growth of GaAs nanoridges consists of three distinct stages. Filling the trench in the first stage exhibits a step-flow growth behavior. Once the structure grows above the mask surface, it enters the second stage of growth by forming {101} side facets as the (100) flat top facet progressively shrinks. In the third stage, the fully formed nanoridge begins to overgrow onto the mask with a significantly reduced growth rate. We develop a kinetic model that accurately describes the width-dependent evolution of the nanoridge morphology through all three stages. MOVPE growth of fully formed nanoridges takes only about 1 min, which is 60 times faster than in our set of molecular beam epitaxy (MBE) experiments reported recently, and with a more regular, triangular cross-sectional geometry defined solely by the {101} facets. In contrast to MBE, no material loss due to Ga adatom diffusion onto the mask surface is observed in MOVPE until the third stage of growth. These results are useful for the fabrication of GaAs nanoridges of different dimensions on the same substrate for various applications and can be extended to other material systems.

## Abstract 2

We outline the growth mechanism and shape evolution of GaAs nanoridges by selective area MOVPE with three well-identified, distinct stages of growth.

## Introduction

Selective area epitaxy (SAE) is a powerful technique that can be used to create high-quality crystalline micro- and nanostructures in lithographically defined locations on a host substrate. The technique relies on the use of a mask layer, usually an amorphous dielectric, with patterned openings in prescribed locations. When growth conditions are properly chosen, crystal growth on the mask is suppressed while growth in the open patterned area is favored.^1^

One advantage of SAE over self-assembled growth techniques is the degree of control over the shape and position of the growing crystal. Simply by changing the geometry of the pattern, the crystal orientation of the substrate, and the growth parameters, one can engineer the morphology of the growing nanostructures.^2−5^ SAE has been used to grow a wide variety of semiconductor nanostructures with different dimensionalities, including quantum dots, vertical and horizontal nanowires, nanowire networks, nanomembranes, and nanofins.^6−12^ Beyond versatility in nanostructure morphology, SAE also benefits from enhanced scalability; wafer-scale patterning allows for the easy creation of devices with different dimensions and complexity on the same substrate.^13^

Of the various possible nanostructures, GaAs nanoridges (sometimes referred to as nanomembranes in other studies) stand to benefit greatly from a deeper understanding of the selective area growth process. Such nanoridges can serve as a buffer layer template for the subsequent growth of nanowires of different materials, with a reduced density of crystal defects.^14^ InAs, InGaAs, and InSb nanowires are of particular interest for applications in quantum computing and infrared (IR) photodetection.^15,16^ Rather than growing these nanowires directly on a patterned substrate, growing them on top of a GaAs nanoridge buffer allows for more efficient elastic strain relaxation and additional geometric confinement.^17^ Additionally, growing the nanowires on raised templates prevents the migration of impurities present on the substrate surface from prior processing steps and allows additional functionalization, such as by remote doping.^18^

To take full advantage of GaAs nanoridges as nanowire templates, it is important to precisely understand their growth, including the underlying mechanisms and factors affecting the growth rate and resulting morphology. GaAs nanomembranes grown by molecular beam epitaxy (MBE) have previously been studied.^11,19,20^ To the best of our knowledge, analogous investigations have not been reported for metal–organic vapor-phase epitaxy (MOVPE)-grown nanoridges on (100) substrates. MOVPE growth phenomena at the macroscopic scale are well documented for GaAs,^21−23^ as is the growth of out-of-plane nanostructures by SAE,^24−26^ but growth in nanoscale trenches to form nanoridges has not yet been properly characterized. Importantly, a few studies have highlighted the influence of temperature and V/III ratio on the resulting morphology and faceting of GaAs nanostructures by MOVPE.^5,27^

In this work, we investigate the growth mechanisms of GaAs nanoridges on exactly-oriented GaAs (100) substrates in selective area openings by MOVPE. Using scanning electron microscopy (SEM) and atomic force microscopy (AFM), we observe layer-by-layer, step-flow growth and identify the primary driving force and geometrical constraints. During the main growth phase, nanostructures are dominated by (101) and (101̅) side facets and a flat (100) top facet, which diminishes as the growth proceeds until the side facets dominate. Islands and terraces are present on this top facet throughout the growth, and their shape is related to the atomic coordination present at the step edge. We study the effects of a high-temperature, in situ deoxidation step on the depth and topography of the exposed GaAs trenches before growth. We find that the GaAs atoms rearrange at the bottom of the trenches, giving rise to a valley-like structure with sloped edges composed of atomic steps. Depending on the geometrical parameters, some trenches experience a net loss of material such that the depth of the trench increases after annealing, whereas some trenches accumulate material. We develop a kinetic model to describe the growth in three distinct phases. Finally, we highlight several important differences between MOVPE and MBE growth and comment on the potential advantages of MOVPE as a growth technique for such GaAs nanostructures.

## Results and Discussion

**Figure 1:**
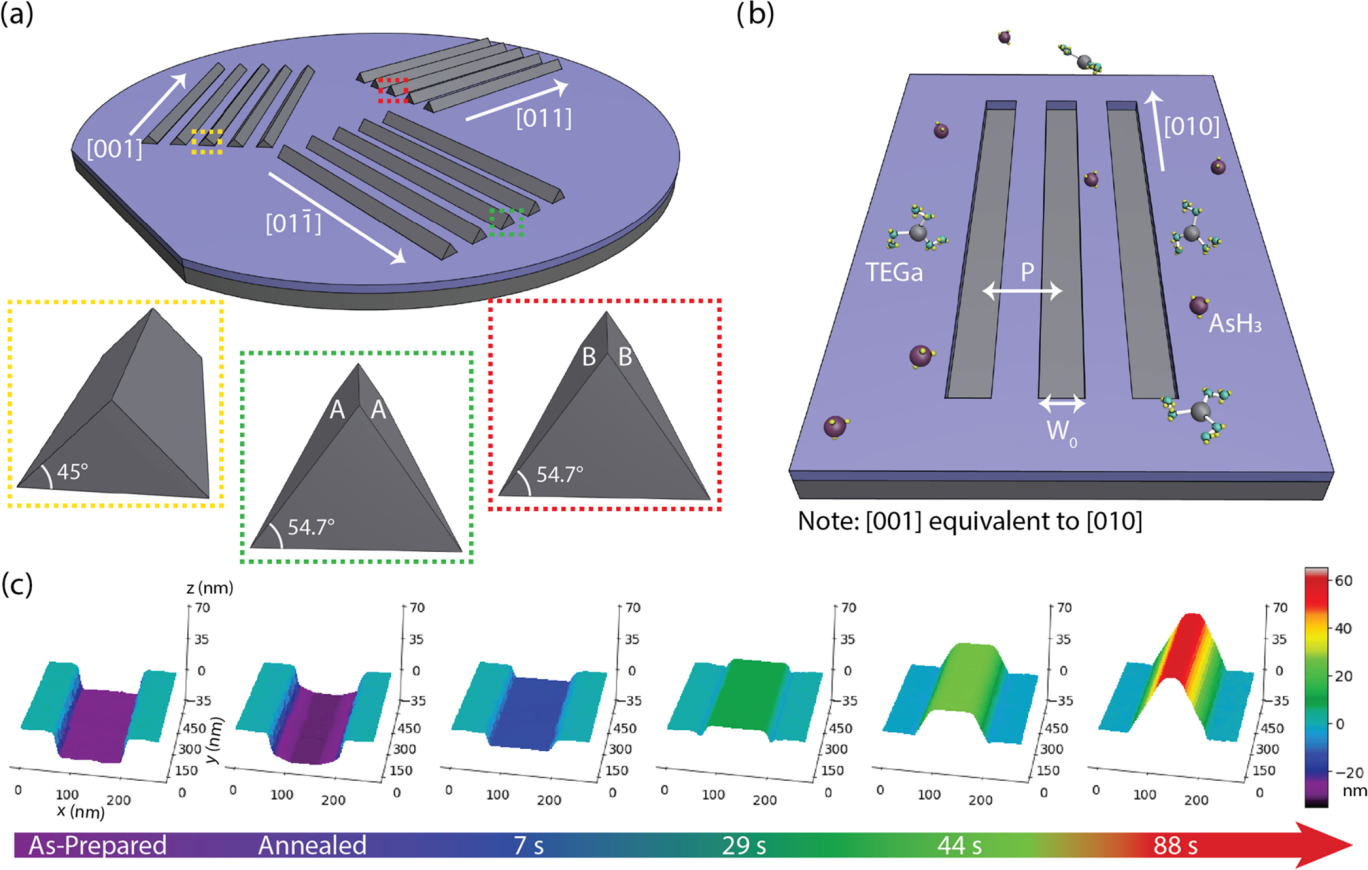
(a) Schematic diagram showing possible orientations for nanoridges on a GaAs (100) substrate, highlighting the different faceting and aspect ratios depending on the crystal direction. [001] and [010]-oriented nanoridges are equivalent and have nonpolar {101} facets, whereas [011̅]-oriented nanoridges have A-polar (Ga-terminated) {111} facets and [011]-oriented nanoridges have B-polar (As-terminated) {111} facets. (b) Schematic diagram showing an ungrown substrate with MOVPE precursors and the main geometrical parameters investigated for the SiO_2_ mask pattern: width (*W*) and pitch (*P*). *W*_0_ represents the nominal width. (c) AFM contour plots showing the time evolution for a growing nanoridge with a nominal width of 100 nm, oriented along the [010] direction.

Figure 1a shows a tilted-view schematic diagram of GaAs nanoridges grown along the high-symmetry directions of a (100) substrate. In general, nanoridges oriented along both ⟨011⟩ and the ⟨001⟩ directions exhibit a triangular cross section, though their facets are defined by different crystal planes. Nanoridges oriented along [011] and [011̅] directions are characterized by polar {111} side facets,^20^ whereas nanoridges oriented along [010] and [001] directions give rise to nonpolar {101} facets. We have investigated the MOVPE growth of nanoridges along each of these crystal directions. For simplicity, here we focus on following the evolution of nanoridges oriented along the [010] direction. See the Supporting Information (SI) for a comparison with growth along the [011̅] direction.

Figure 1b shows a schematic representation of the nanostructured pattern on the GaAs substrate, featuring elongated trenches in an oxide mask. The pattern consists of arrays of 10 trenches, whose geometry is characterized by their width (*W*) and pitch (*P*). Pitch represents the center-to-center distance between neighboring trenches. The length of the structures is fixed at 50 μm. The present work has investigated nominal pitches of 500, 1000, 2000, and 4000 nm and nominal widths of 100, 160, and 240 nm. In the fabricated substrates, the actual width of the trenches can deviate significantly from the nominal width, referred to as *W*_0_ from here on, due to process nonidealities such as overexposure and proximity effect during electron-beam lithography (EBL) and etching. Unless otherwise stated, values presented here refer to the nominal width.

Figure 1c shows three-dimensional (3D) reconstructions of atomic force microscopy (AFM) measurements from a growth time series, displaying how the GaAs growth develops for trenches of width 100 nm and pitch 1000 nm. Growths were performed at 650 °C using TEGa and arsine with a nominal V/III ratio of 3.2 and a planar growth rate of 1 nm/min. We observe that an initial thermal annealing at 820 °C under arsine induces a morphological change at the exposed GaAs surface, forming a valley-like structure with atomic steps at the edges. Upon initiation of the growth stage via the introduction of triethyl gallium (TEGa), the nanostructure forms a flat (100) top facet, filling in the post-anneal “valley”. After sufficient time, the nanostructure emerges above the level of the SiO_2_ mask, at which point the (100) top facet begins to shrink in area as (101) and (101̅) side facets appear, generating a trapezoidal cross section. Throughout the growth process, [100] remains the fastest growth direction, meaning the nanoridges grow primarily upward, with minimal lateral growth. The growing nanoridge is thus constrained by the patterned SiO_2_ mask and the slower growing {101} side facets until the nanoridge is fully formed and the cross section becomes roughly triangular. After achieving its final shape, the nanoridge does continue to grow slowly outward in all directions, maintaining the same morphology dominated by (101) and (101̅) facets.

**Figure 2:**
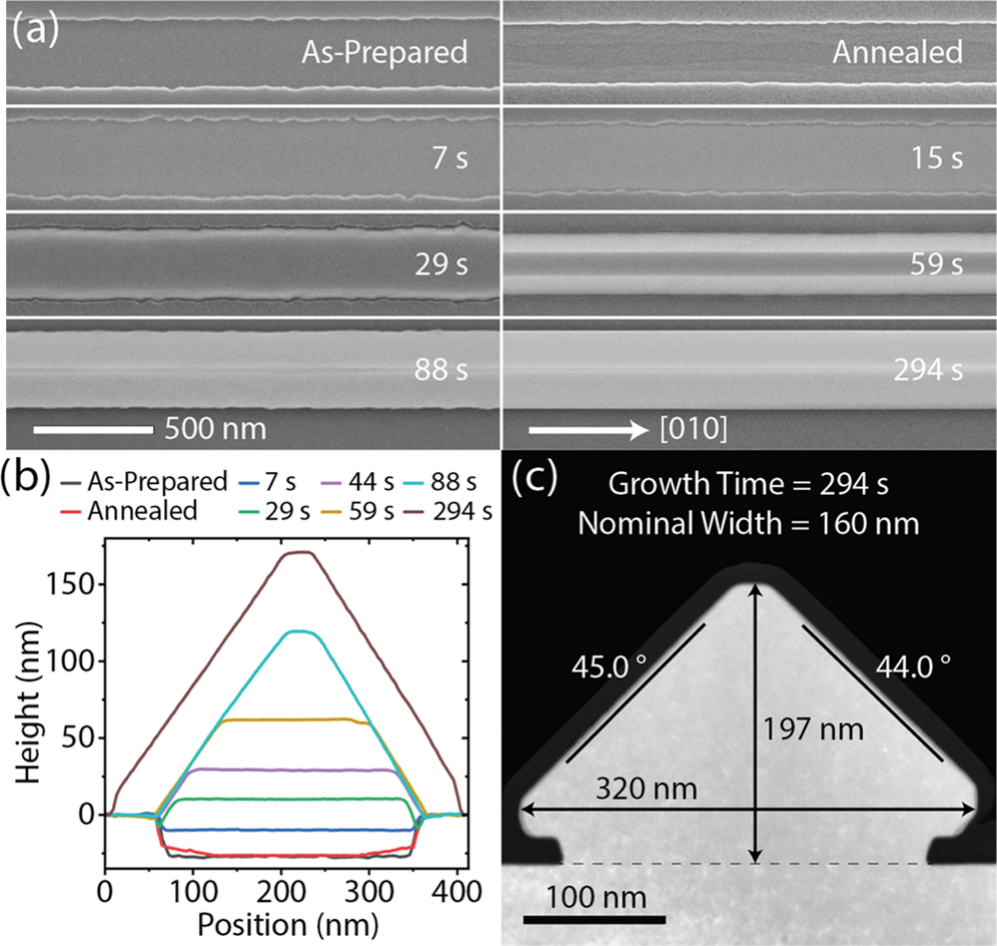
(a) Top-view SEM images of nanoridges at various stages of growth. The variation in width is inherent to the substrate preparation, including EBL and etching. The nominal width is 200 nm for each trench. (b) AFM profiles for different growth times for a nanoridge with a nominal width of 240 nm. (c) Cross-sectional HAADF-STEM image of a nanoridge with measurements of height, width, and facet angles relative to the substrate. Note that the axes in (b) are not to scale, so the aspect ratio appears different compared to (c), which shows the true proportions.

Figure 2 gives a more detailed view of the growth evolution described above. Figure 2a shows top-view SEM images of the time series, including the as-prepared substrate before annealing, after annealing and growth times up to 294 s. One may observe a small amount of roughness at the bottom of the trench in the as-prepared substrate. This is likely the result of inhomogeneities in the etching process. After annealing, one observes step-like features that run along the length of the trench, forming a valley down the middle. The height of these steps is roughly atomic in nature. See the SI for a more detailed view. The shortest growth time studied corresponds to 7 s, after which the GaAs layer appears to adopt a flat (100) top facet. After 29 s, the growing GaAs nanoridge has emerged above the level of the oxide mask and begins to develop (101) and (101̅) side facets. By 88 s, even the widest nanoridges have achieved their final morphology, characterized by almost exclusively {101} facets, except for the very top of the ridge where the side facets meet, which appears slightly rounded.

Figure 2b shows AFM cross sections that have been taken for 240 nm nominal width nanoridges at various growth times, allowing for a more precise understanding of their morphological evolution over time. Here too we observe the valley-like nature of the trench after annealing and the flat (100) top facet of the developing nanoridge, which shrinks in area after the structure grows above the level of the oxide mask, while the (101) and (101̅) facets become more pronounced. In Figure 2c, we see a cross-sectional high-angle annular dark-field scanning transmission electron microscopy (HAADF-STEM) image showing the final form of a nanoridge, which was grown for a full 294 s. The image is taken along the [010] zone axis. The main facets of the nanostructure have close to 45° angles with the substrate, as expected for {101} facets.

Interestingly, the HAADF-STEM cross section provides insight into the full morphology of the nanoridges, beyond what is accessible by AFM and top-view SEM. In Figure 2c, we observe that the nanoridge is not exclusively defined by the (101) and (101̅) facets, but also by what appear to be additional facets at the far edge of one side of the nanoridge as well as some rounding at each of the corners. The nanoridges exhibit a small amount of lateral growth in the [001] and [001̅] directions, above the oxide mask. The growth rate in this direction is low, as this lateral growth only becomes significant once the (101) and (101̅) facets have developed and never approaches the growth rate observed when the flat (100) facet is present. This will become clear later, when we model the height, width, and grown volume of these structures as a function of growth time.

**Figure 3:**
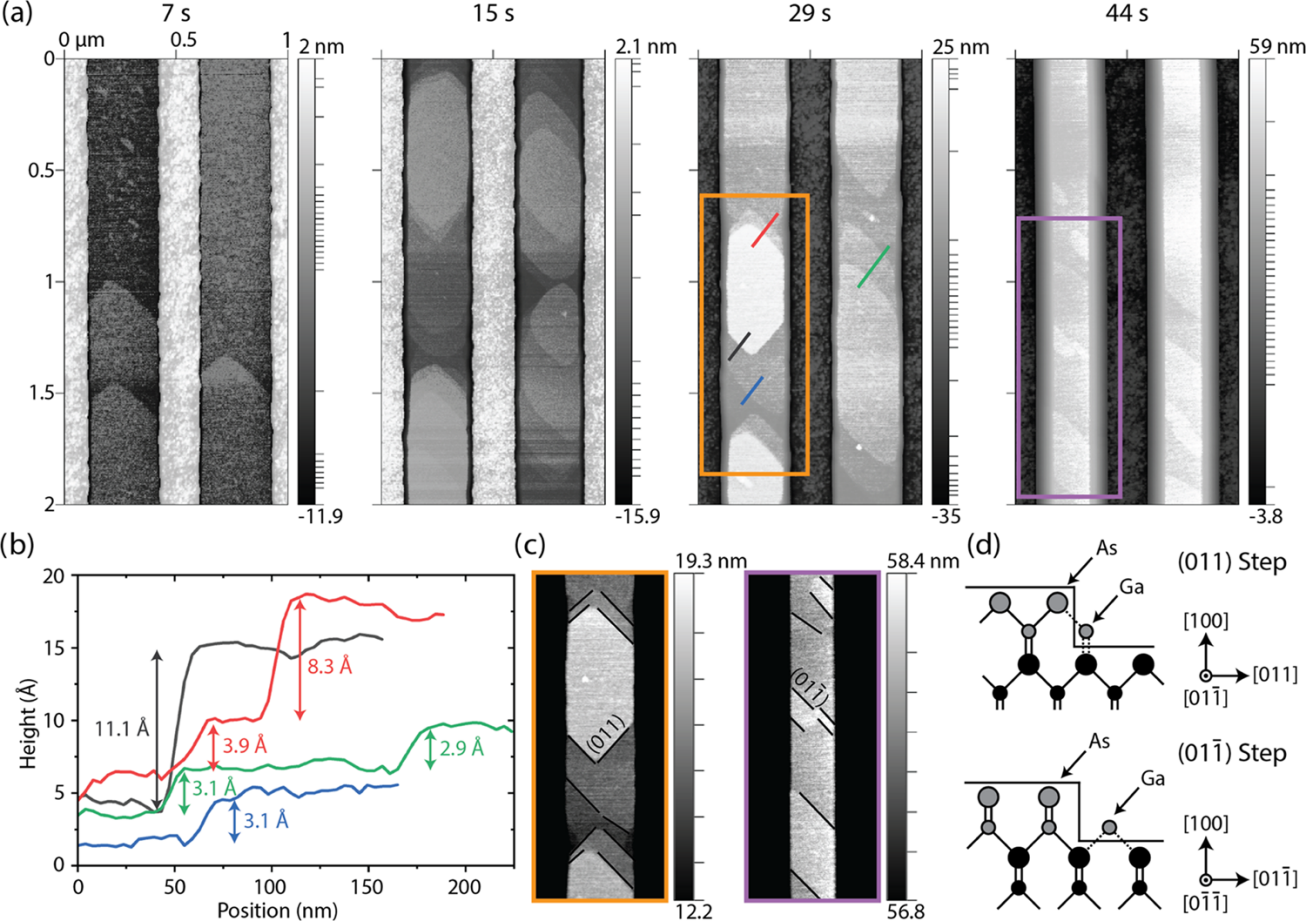
(a) AFM contour maps showing the formation of atomic steps and islands for every stage of growth where the (100) surface is still present. An adaptive nonlinear coloring has been adopted to better highlight the nanoscopic texture at multiple heights. (b) Line scans taken from the 29 s growth as indicated by the colored lines in (a), showing the atomic nature of the observed terraces. (c) AFM contour maps for the 29 and 44 s growth, respectively, as indicated by the colored boxes in (a). Black lines indicate the presence of steps oriented parallel to (011) and (011̅) planes. The height scale has been adapted to show only the top surface of the growing nanoridge. (d) Schematic illustration showing the atomic coordination of Ga and As atoms for (011) and (011̅)-oriented steps. Gray circles represent the growing monolayer, whereas black circles are the underlying layer. The size of the circle corresponds to the atomic species.

We turn our attention to the nature of the growth in the [100] direction while the flat (100) top facet is present. Figure 3a shows high-resolution AFM contour plots of nanoridges with a pitch of 500 nm and a nominal width of 240 nm at various stages of growth. From the earliest stages of growth, what appear to be atomic steps are observed on the top facet, which develop into islands that can be over a micrometer in length. As the growth continues, the islands do not become more distinct from one another, indicating that the lower layers grow outward and merge together. Thus, at any given time, the islands appear to be only a few monolayers tall, as their lower levels are continually coalescing. This behavior resembles step-flow growth, which has been observed for on-axis GaAs (100) wafers as described by Orme et al.^28^ The wafers used in this study are not intentionally misoriented, and because the step flow does not occur in only one direction, it appears that this island step-flow growth is a spontaneous result of the growth conditions and not a result of an unintentional misorientation of the growth substrate.^29^

The presence of these coalescing islands is evidence of the competing effects of nucleation and step-flow growth. The height and lateral dimension of the islands along the length of the trench should be a function of the nucleation rate, the Ga diffusion length, and the energetic barrier for Ga adatoms to hop down from one monolayer to the one below, known as the step edge or Ehrlich–Schwoebel barrier.^28^ Interestingly, this result is markedly different from similar growths observed by MBE, where growing islands are much taller, exhibiting distinct faceting and with no signs of step-flow growth.^20^

Figure 3b shows various line scans taken perpendicular to the island edges as indicated in Figure 3a. Although our measurements are limited in their accuracy due to noise, it is clear that the smallest steps are around 3 Å in height, which roughly corresponds to half of the lattice constant of GaAs (5.65 Å), or one monolayer of GaAs in the [100] direction. Some terraces also appear to be multiple monolayers thick, a result of step bunching. For example, in Figure 3b, the step height measuring 8.3 Å could correspond to 1.5 unit cells (∼8.5 Å, 3 monolayers) and the step height of 11.1 Å could be 2 full GaAs unit cells (∼11.3 Å, 4 monolayers). One should note that the samples are exposed to atmosphere after growth, so any GaAs surface is expected to be oxidized, though this is not expected to impact the measurements significantly.^30^

The shape of the islands also provides insight into the nanoscale processes occurring during growth. In Figure 3a, one immediately notices the asymmetric front of the islands and terraces. Looking more closely, as in Figure 3c, we observe that in the early stages of growth, there are two main growth fronts which are perpendicular to each other, forming an average angle of 46.5 ± 2.4° with the trench sidewalls. Based on the known symmetry of GaAs, it appears that these fronts correspond to edges oriented along (011) and (011̅) surfaces. As the growth proceeds, the (011)-oriented edges seem to shrink, until the observed growth fronts almost exclusively correspond to the (011̅)-oriented edges, with a measured angle of 41.7 ± 4.8°. This implies anisotropic step motion, which is described for GaAs (100) surfaces by Asai et al.^31^ (011)-oriented steps provide three bonds to an arriving Ga adatom. In contrast, (011̅)-oriented steps provide only two bonds to Ga. The two cases are schematized in Figure 3d. Thus, (011) step-flow velocity is higher under the MOVPE conditions used here, giving rise to the observed island shape and elimination of (011) step edges.

**Figure 4:**
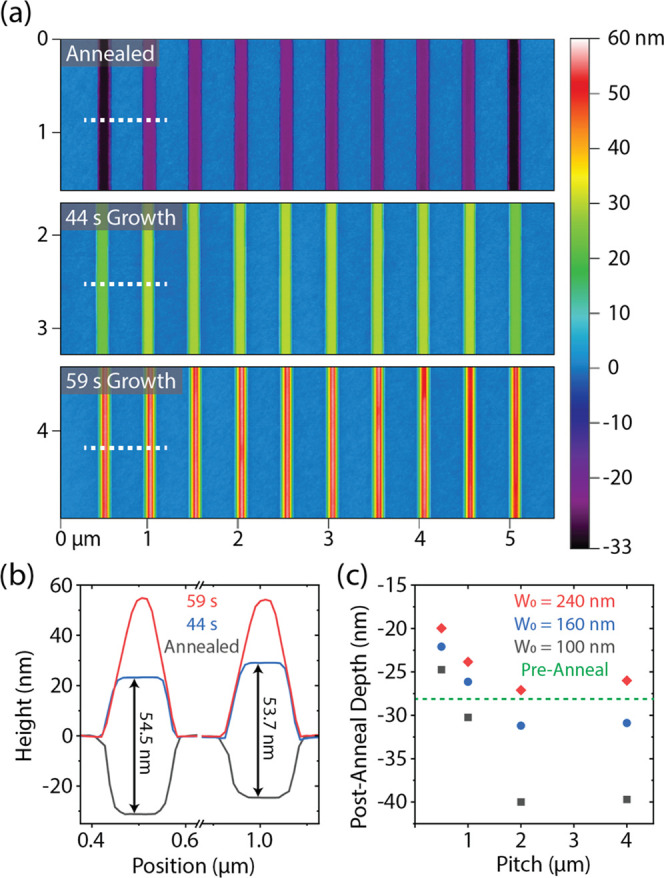
(a) AFM contour maps for a full array of 10 nanoridges with a nominal width of 100 nm at three different stages of growth. (b) AFM profiles of the outermost and neighboring trench as indicated by the dashed white line in (a) showing that after annealing the outside trench is deeper than other trenches in the array, but that the growth rate for all trenches is roughly equal, resulting in nanostructures of similar height once the {101} facets are fully developed after 59 s. (c) Plot showing the measured height of trenches after annealing for different nominal widths and pitches. All measurements were taken from trenches near the center of the array. The dashed green line indicates the average height for all trenches before annealing (experimental data given in the SI).

Next, we turn our attention toward the impact of geometrical constraints on the growth. To do this, we first consider whether growth occurs homogeneously across all parts of a given array of nanoridges. Figure 4a shows AFM contour plots for arrays of 10 nanoridges with nominal width of 100 nm and pitch of 500 nm. Three different growth stages are represented: after annealing, 44 s of growth and 59 s of growth. Figure 4b shows line scans from the two leftmost trenches. After annealing, the outermost trench is deeper compared to the inner trenches of the array. This is observed both on the left and right sides of the array, while no significant height difference is observed among the inner trenches. This observation is attributed to a net loss of material from the outside trenches due to desorption of the GaAs during annealing. When temperatures are high enough to cause decomposition of the GaAs, As readily desorbs into the vapor phase, whereas Ga adatoms remain adsorbed for some time and are free to diffuse on the surface. These adatoms not only diffuse on the surface of the GaAs but can also diffuse out of the trenches onto the oxide mask.^32^ Ga adatoms on a GaAs surface can either desorb or be recaptured by the GaAs if a corresponding As atom is provided from the reactor atmosphere, which is supplied with arsine during the annealing. Ga adatoms on the oxide mask, however, can only be desorbed, unless they ultimately diffuse back into a trench to rejoin the GaAs surface. We neglect the possibility of GaAs nucleation on the mask since the growth has proven to be very selective in nature.

For sufficiently high temperatures, such as during annealing, an adatom can leave one trench, diffuse across the mask, and enter another to be reincorporated into the GaAs surface. Thus, material sharing occurs between neighboring trenches. The amount of material diffusing out of the trench should be independent of its number of neighbors. However, the amount of Ga diffusing into the trench should be proportional to its number of neighbors (1 or 2 for linear structures in an array, 0 for isolated trenches), and this effect should be stronger for arrays with a smaller pitch, i.e. trenches which are closer to each other. This effect is apparent in Figure 4c, where it is shown that narrow trenches with larger pitches lose GaAs material during annealing, whereas wide trenches with small pitches actually gain material during annealing. This accumulation of material for certain array geometries is attributed to a small background of Ga in the reactor, left over from previous growths. This has been confirmed using similarly patterned Si substrates, which show small amounts of GaAs growth inside their trenches after annealing under the same conditions. All trenches are supplied with a small amount of Ga from the reactor background, but arrays of narrow trenches with larger pitches lose more material than they gain from the background supply, resulting in a net loss of material. Larger trenches which are closer together are able to “recycle” material lost from their neighbors, mitigating their own material loss and allowing for some amount of growth during the arsine anneal, even though the TEGa supply during this step is zero. Material loss during annealing has also been observed in MBE, with similar trends for width and pitch dependence.^20^

Using the depth measurement after annealing, we can estimate that the effective diffusion length of Ga on the SiO_2_ mask is somewhere between 1 and 2 μm for the annealing conditions, similar to what was previously reported by Sobanska et al.^33^ For larger pitches, we conclude that there is no material sharing between neighboring trenches. Instead, any Ga which is liberated from the substrate during annealing is likely to be desorbed and lost, resulting in the asymptotic behavior we observe in Figure 4c for large pitches. We also conclude there is little to no difference in growth rate for trenches at the edge or center of an array for the main growth step; both trenches shown in Figure 4b grow nearly the same amount in the first 44 s of growth and end up with the same height once the (101) and (101̅) facets are fully formed. This is consistent with the observation that pitch has little influence on the growth rate (see the SI for more details). Thus, nearest neighbors likely do not share Ga during this main growth step, contrary to what has been observed for MBE growth of comparable structures.^20^ This could indicate that, for the given growth conditions, Ga adatom mobility is insufficient for this material-sharing process, or that the material contribution from this phenomenon is much less than that of the direct supply from TEGa.

## Modeling

**Figure 5:**
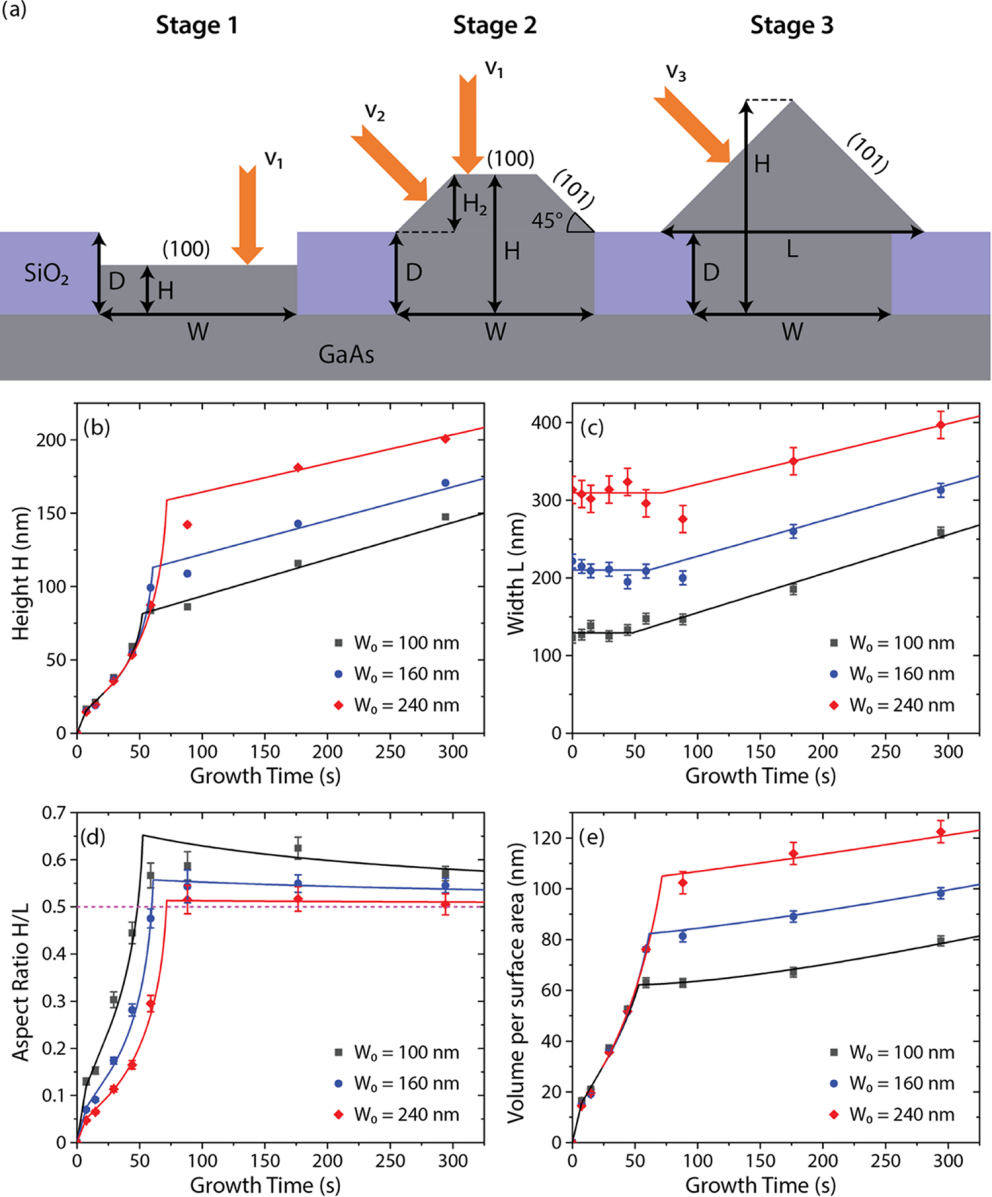
(a) Illustration of the different growth stages of a GaAs nanoridge in a trench of width *W* and depth *D*. The height *H* is measured from the bottom of the trench. The width of the nanoridge equals *W* in stages 1 and 2 but becomes larger than *W* when the nanoridge extends onto the mask in stage 3. The attachment rate of the top (100) facet is *v*_1_. The attachment rates of the side {101} facets in stages 2 and 3 are *v*_2_ and *v*_3_, respectively. Morphology of the nanoridges is presented in terms of (b) maximum height, (c) maximum width, (d) aspect ratio, and (e) volume per maximum surface area, or mean height of the structure. Symbols show the kinetic data for nanoridges grown for different times in 1000 nm pitch arrays of trenches having nominal widths *W*_0_ of 100, 160, and 240 nm. Lines show the fits by the model with the parameters summarized in Table 1. The dashed line in (c) shows the asymptotic aspect ratio α = 1/2 at large *L*.

To understand and quantify the growth kinetics of GaAs nanoridges in selective area MOVPE, we have established the following model. The growth is divided into three stages, which follow from the data in Figure 2b. The growth stages and the corresponding geometries are illustrated in Figure 5a. Stage 1 proceeds via layer-by-layer two-dimensional (2D) growth in the mask opening until the trench is fully filled. In stage 2, the incomplete nanoridge has the shape of a truncated triangle restricted by the two equivalent {101} side facets and the (100) top facet. This stage continues until the nanoridge is fully formed, corresponding to the disappearance of the (100) facet on the top. In stage 3, the nanoridge extends onto the mask surface, maintaining the triangular shape with (101) and (101̅) side facets. The growth is assumed Ga-limited, as usual in SAE.^19,20,32,33^ In each stage, the MOVPE growth rate of the nanoridge volume per unit length, or the cross-sectional area *S*, is given by the standard expression^34^
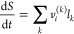
1Here, *l*_*k*_ are the lengths of facets *k* which grow in stage *i* by attaching Ga atoms at the rates *v*_*i*_^(*k*)^(nm/s) in this stage. These growth rates include the desorption of Ga adatoms from different GaAs surfaces. At a given geometry, the only unknown parameters of the model are the growth rates in different stages, but they can be precisely determined from the kinetic data on the morphological evolution of nanoridges. Therefore, our model essentially contains no fitting parameters. No noticeable pitch dependence of the morphological evolution was observed for nanoridge arrays with pitches ranging from 500 to 4000 nm, as previously discussed. Therefore, the growth rates *v*_*i*_^(*k*)^ do not include the pitch-dependent diffusion flux of Ga adatoms from or onto the mask, which is effective in MBE growth.^20,32^

For the geometries shown in Figure 5a, the nanoridge structure is fully characterized by its height *H* (measured from the bottom of the trench) and width *L*, which equals *W* in stages 1 and 2, and gets larger than *W* in stage 3. In stage 1, the rectangular structure has the surface area *S* = *WH*, and the only facet exposed to vapor is the top (100) facet of constant length *l*_1_ = *W*. Based on the data in Figure 5b,c, the height grows faster in the first 7.4 s, which is probably related to the surface roughness or polynuclear growth in the bottom of the trench, which improves sticking of GaAs. Introducing the growth rate *v*_1_^*^ in the bottom of the trench and *v*_1_ in a later step, from eq 1, we have
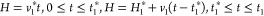
2Here, *t*_1_^*^ = 7.4 s is the end of the fast growth step in the bottom of the trench and *t*_1_ is the end of stage 1 where *H*(*t*_1_) = *D*.

In stage 2, the 3D structure grows out of the trench, starting from the planar layer and developing into a truncated triangle restricted by the top (100) facet and the two equivalent {101} side facets, which make an angle of 45° to the substrate surface. The top (100) facet progressively shrinks to complete the full triangle restricted solely by the (101) and (101̅) side facets. From geometry, we have *H* = *D* + *H*_2_ and *S* = *WD* + *H*_2_(*W* – *H*_2_), where *H*_2_ = *H* – *D* is the height of the structure above the mask surface. The length of the top facet *l*_1_ equals *W* – 2*H*_2_. The length of the two side facets *l*_2_ equals , with . The attachment rate *v*_1_ on the top facet should be the same as in stage 1. We refer here to *v*_1_ as an attachment rate rather than growth rate because, as will be described later, Ga diffusion from one facet to another becomes important in this stage. The attachment rate refers only to the rate that Ga attaches to each facet from the gas phase, neglecting surface diffusion. Introducing the attachment rate of the two side {101} facets *v*_2_, eq 1 gives
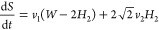
3

Using the above equation for *S*, we obtain a nonlinear equation for *H*_2_, the only parameter which changes in stage 2
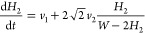
4with the initial condition *H*_2_(*t*_1_) = 0. The solution is given by
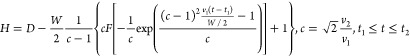
5Here, the Lambert function *F*(*z*) is defined as the root of an equation *F* exp(*F*) = *z*. Stage 2 continues until the moment in time *t*_2_, where *H*_2_(*t*_2_) = *W*/2. In fact, the transition from stage 2 to stage 3 occurs slightly earlier than the formation of the full triangle with a sharp tip on top, consistent with the slightly rounded tips seen in Figure 2. This feature is indeed reproduced by the fits and confirms the well-known avoidance of a sharp edge in crystal growth.^34^

Stage 3 begins when the fully formed nanoridge starts extending onto the mask surface, maintaining its triangular shape. The only parameter which changes in this stage is the width of the triangle *L*. The surface area *S* = *WD* + *L*^2^/4 changes by the Ga flux impinging onto the two {101} side facets with length . The height of the structure including the root is *H* = *D* + *L*/2. Hence, the growth equation is given by
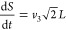
6where *v*_3_ is the attachment rate of the side facets in this stage. As discussed above, the growth slows significantly in stage 3, which is why *v*_3_ is expected to be lower than *v*_2_. Using the above equation for *S*, we arrive at the linear growth regime
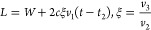
7

The nanoridge height in this stage is given by *H* = *D* + *L*/2. In principle, stage 3 continues until the nanoridges merge, but a time-independent growth rate *v*_3_ throughout the whole process is not guaranteed.

Although the nanoridge structures are fully described by the two parameters *H* and *L* for a given geometry in each stage, two other parameters may be of interest. The aspect ratio is defined as the ratio of the maximum height over the maximum width of the structure, that is
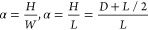
8in stages 1, 2, and in stage 3, respectively. The total volume of the structure per specific surface area, or the mean height, is defined according to
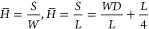
9in stages 1, 2, and in stage 3, respectively. Here, we choose the maximum surface area (corresponding to the maximum width) as the specific surface area of the nanoridge. The total volume can alternatively be presented per surface area of the trench (corresponding to a constant width *W*), as done by Dede et al.^20^ The difference between the two representations appears only in stage 3 where *L* > *W*.

Figure 5b,c shows the kinetic data for the maximum height *H* and width *L* of GaAs nanoridges grown in 1000 nm pitch arrays with three different nominal widths of the trenches: 100, 160, and 240 nm. The data were obtained from statistical analysis of the samples grown for different times between 7.4 and 294 s. Figure 5c,d shows the aspect ratios and mean heights calculated from the measured *H* and *L* using eqs 8 and 9. Lines in Figure 5b–e represent the fits obtained from eqs 2, 5, and 7 in different stages. All of these curves were obtained with *v*_1_^*^ = 2.1 nm/s in the fast step and *v*_1_ = 0.85 nm/s in the slow step of stage 1 and in stage 2, *t*_1_^*^ = 7.4 s and *t*_1_ = 24.2 s, yielding a mean depth *D* of all trenches of 29.8 nm. In stages 1 and 2, we used the average values of the measured actual widths given in Table 1. The times *t*_2_ corresponding to the end of stage 2, and the {101} attachment rates in stages 2 and 3 are also given in Table 1.

**Table 1:** Parameters of GaAs Nanoridges Grown in Differently Sized Trenches

nominal width *W*_0_ (nm)	average actual width *W* (nm)	end of stage 2 *t*_2_ (s)	{101} attachment rate in stage 2 *v*_2_ (nm/s)	{101} attachment rate in stage 3 *v*_3_ (nm/s)
100	129.2	50.1	1.26	0.177
160	210.0	61.0	2.12	0.162
240	309.4	71.6	2.55	0.138

The morphological evolution of nanoridges shown in Figure 5b–e reveals the following trends. Linear increase of the height in the short stage 1 is followed by a highly nonlinear behavior in stage 2, which is explained by progressive shrinking of the top facet. The vertical growth rate becomes very high as the facet shrinks, approaching a sharp tip on top. Indeed, the growth rate tends to infinity at *H*_2_ → *W*/2 according to eq 4. As mentioned above, none of the fitting curves in Figure 5b reach the vertical asymptote, revealing that the nanoridge tips remain slightly rounded. After forming the triangle shape, the nanoridges start extending onto the mask surface in stage 3, where the width of the structures increases beyond the trench width. This growth stage is linear in time according to Figure 5b,c. The aspect ratios in Figure 5d reach their maxima at the end of stage 2 and then slowly decrease to the asymptotic value of 1/2 corresponding to negligible contribution of the buried root at large *L*. The total volume per surface area in Figure 5e increases super-linearly in stage 2 and linearly in stage 3.

According to the observed growth features and the corresponding parameter values in Table 1, the growth of fully formed nanoridges takes longer time in wider trenches, which is expected since forming a larger nanoridge requires more material to be grown. The {101} attachment rates in stage 2 depend on the width, and are systematically larger than the (100) growth rate of 0.85 nm/s. This is also expected because the stable (100) crystal plane should be the slowest growing facet, which explains the stable (100) growth orientation in stage 1. Without any oxide mask, this orientation would remain in a later growth step. However, extending the nanoridge onto the mask surface requires the formation of an energetically costly interface between GaAs and oxide. To avoid this interface on surface energetic grounds, the {101} side facets are formed and the 3D nanoridge geometry developed. GaAs material is attached faster from the sides than from the top, but then redistributed by surface diffusion from the side facets to the top to keep the constant width of the truncated structure without lateral extension onto the mask. This becomes impossible in stage 3, where the GaAs volume can only increase by extending the nanoridge width. Finally, the growth rate in stage 3 is significantly reduced. This effect should be due to additional desorption of Ga from the {101} side facets, probably via diffusion onto the mask surface. This diffusion, however, is independent of pitch and only slightly depends on the width, which is evidenced by very similar slopes of the linear curves in Figure 5b,c in stage 3. The growth suppression in stage 3 should be driven by surface energetics, similar to stage 2.

The model reproduces the experimental data very well. The deviations seen in Figure 5b,c for heights and widths are explained by (i) tolerances in lithographic patterning process and (ii) variations in GaAs deposition rates from sample to sample. For instance, the width data in Figure 5c vary in stages 1 and 2 because they are obtained from measurements of different nanoridges, while they should stay constant in the modeling curves that describe the growth kinetics of one nanoridge. For the relative values such as the aspect ratio in Figure 5d and volume per surface area in Figure 5e, the correlation of the model with the data is better. Any variation in the deposition rate almost cancels in the aspect ratio, where both height and width are roughly proportional to *v*_1_. The volume per surface area almost circumvents the uncertainty in the trench width *W*, because it is normalized to unit width. Without surface diffusion of Ga adatoms, this growth behavior should pertain to any material which has a stable (100) growth orientation at the beginning and stable {101} side facets at the end. Similar growth kinetics and morphologies should be observed for nanoridges of different depths and widths, which only influence the durations of stages 1 and 2.

MOVPE growth of fully formed nanoridges takes only 50 s in 100 nm wide trenches and 72 s in 240 nm wide trenches, regardless of the pitch. The same process takes at least 55 min in MBE for the conditions reported previously, where narrower trenches are filled slower due to negative Ga diffusion from trenches onto the mask surface.^20^ Our Ga-limited (100) growth rate equals 0.85 nm/s according to the data. The maximum MBE GaAs (100) growth rate reported by Dede et al.^20^ is ∼0.03 nm/s, which is 28 times lower than here. The MOVPE growth time of fully formed nanoridges is 60 times shorter than in MBE. The additional factor of about 2 should be due to the surface diffusion of Ga away from the trenches,^20^ which is present in MBE and absent in MOVPE. In fact, MOVPE experiences a significant growth rate enhancement for SAE compared to planar thin film growth, whereas MBE experiences a growth rate reduction that is highly pattern-dependent. Significantly higher growth rates in MBE are likely not possible due to loss of selectivity. Furthermore, MOVPE-grown nanoridges develop no high-index facets such as {311} facets reported by Dede et al.^20^ Therefore, the MOVPE technique has some advantages over MBE for obtaining GaAs nanoridges, including a much faster growth process, more regular faceting at intermediate stages of growth, and simple, quasi-self-limiting growth kinetics enabling well-controlled geometries of different dimensions on the same substrate. These properties could be desirable to create smooth buffer layers for subsequent nanowire growth without the need for a surfactant.^35^

## Conclusions

We investigated the growth of GaAs nanoridges on (100) GaAs substrates by SAE using MOVPE and demonstrated that the growth occurs in three distinct stages. Before the growth begins, an annealing step results in the rearrangement of the GaAs surface inside of the trench, forming a valley-like structure with atomic terraces. Depending on their width and proximity to neighboring trenches, some trenches experience a net loss of material, becoming deeper, whereas others appear to gain material, a result of residual Ga in the MOVPE reactor from previous growths. During the first stage of growth, the nanostructure is constrained only by the SiO_2_ sidewalls, maintaining a flat (100) top facet. From the earliest stages of growth, this (100) facet develops islands and terraces which are one to several monolayers thick and exhibit a step-flow growth behavior. Once the nanostructure grows above the level of the oxide mask, it enters the second stage of growth, where it begins to develop {101} side facets as the (100) top surface shrinks in area. The final stage of growth begins once the (100) top facet disappears completely. The nanoridges extend onto the mask surface, at which point their growth rate is significantly reduced. We developed a kinetic model to describe this process and determine the primary parameters of the growth for each stage. The growth reported here stands in stark contrast to that observed for MBE, where faceted nuclei develop and coalesce, never forming a flat top facet unless a surfactant is used. Furthermore, the formation of nanoridges with well-defined geometry takes only 50–72 s for trench widths of 100–240 nm, which is 60 times faster than in MBE. No loss of material due to negative diffusion of Ga adatoms from the nanoridges to the mask is observed in MOVPE growth stages 1 and 2, while in MBE, this effect is very pronounced from the beginning of growth. It leads to morphological inhomogeneities and increases the formation time of the full nanoridges by a factor of 2. Overall, we showed that MOVPE is a robust, controllable method for producing nanoridges of different dimensions on the same growth substrate, which is crucial for later functionalization of these structures, such as using them as a template for InAs or InSb nanowire growth.

### Methods

Semi-insulating (100) GaAs substrates were first coated with 27 nm of SiO_2_ by plasma-enhanced chemical vapor deposition (PECVD). Trenches of various widths and pitches were patterned by electron-beam lithography (EBL), using ZEP as a positive tone resist and cold *n*-amyl acetate development. The pattern was transferred to the SiO_2_ using reactive ion etching (RIE) with CHF_3_/SF_6_ chemistry. The resist was then stripped by oxygen plasma before a final etch with dilute buffered oxide etch (BOE) to remove any oxide residues from the bottom of the trenches. The wafer was then cleaved into quarters and transferred to the Aixtron AIX 200 MOVPE system. The growth recipe begins with an annealing step at ∼820 °C under arsine and nitrogen atmosphere. The main growth step takes place at 650 °C with a TEGa molar flow rate of 5.6 × 10^–4^ mol/min and an arsine molar flow rate of 1.8 × 10^–3^ mol/min with nitrogen as a carrier gas for a total gas flow of ∼7 slm. These conditions correspond to a 2D equivalent growth rate of ∼1 nm/min. Various growth times were investigated in this study: 0 (anneal-only), 7.4, 14.7, 29.4, 44.1, 58.8, 88.2, 176.5, and 294.1 s. Growth times stated in the text have been rounded to the nearest whole number. A Bruker FastScan system was used for AFM characterization with ScanAsyst Fluid+ probes.

## Supporting Information Available

Additional details showing growth in [010] vs [011̅]-oriented trenches, atomic steps after annealing, AFM data processing and measurement specifications, depth of trenches before annealing, and width and height data for different pitches (PDF)

## References

[ref1] Aseev P.; Fursina A.; Boekhout F.; Krizek F.; Sestoft J. E.; Borsoi F.; Heedt S.; Wang G.; Binci L.; Martí-Sánchez S.; Swoboda T.; Koops R.; Uccelli E.; Arbiol J.; Krogstrup P.; Kouwenhoven L. P.; Caroff P. Selectivity Map for Molecular Beam Epitaxy of Advanced III-V Quantum Nanowire Networks. Nano Lett. 2019, 19, 218–227. 10.1021/acs.nanolett.8b03733. 30521341 PMC6331184

[ref2] Wang N.; Yuan X.; Zhang X.; Gao Q.; Zhao B.; Li L.; Lockrey M.; Tan H. H.; Jagadish C.; Caroff P. Shape Engineering of InP Nanostructures by Selective Area Epitaxy. ACS Nano 2019, 13, 7261–7269. 10.1021/acsnano.9b02985. 31180645

[ref3] Yuan X.; Pan D.; Zhou Y.; Zhang X.; Peng K.; Zhao B.; Deng M.; He J.; Tan H. H.; Jagadish C. Selective Area Epitaxy of III-V Nanostructure Arrays and Networks: Growth, Applications, and Future Directions. Appl. Phys. Rev. 2021, 8, 021302.

[ref4] Lee J. S.; Choi S.; Pendharkar M.; Pennachio D. J.; Markman B.; Seas M.; Koelling S.; Verheijen M. A.; Casparis L.; Petersson K. D.; Petkovic I.; Schaller V.; Rodwell M. J. W.; Marcus C. M.; Krogstrup P.; Kouwenhoven L. P.; Bakkers E. P. A. M.; Palmstrøm C. J. Selective-Area Chemical Beam Epitaxy of in-Plane InAs One-Dimensional Channels Grown on InP(001), InP(111)B, and InP(011) Surfaces. Phys. Rev. Mater. 2019, 3, 084606 10.1103/PhysRevMaterials.3.084606.

[ref5] Ikejiri K.; Noborisaka J.; Hara S.; Motohisa J.; Fukui T. Mechanism of Catalyst-Free Growth of GaAs Nanowires by Selective Area MOVPE. J. Cryst. Growth 2007, 298, 616–619. 10.1016/j.jcrysgro.2006.10.179.

[ref6] Birudavolu S.; Nuntawong N.; Balakrishnan G.; Xin Y. C.; Huang S.; Lee S. C.; Brueck S. R. J.; Hains C. P.; Huffaker D. L. Selective Area Growth of InAs Quantum Dots Formed on a Patterned GaAs Substrate. Appl. Phys. Lett. 2004, 85, 2337–2339. 10.1063/1.1792792.

[ref7] Vukajlovic-Plestina J.; Kim W.; Dubrovski V. G.; Tütüncüoğlu G.; Lagier M.; Potts H.; Friedl M.; Fontcuberta i Morral A. Engineering the Size Distributions of Ordered GaAs Nanowires on Silicon. Nano Lett. 2017, 17, 4101–4108. 10.1021/acs.nanolett.7b00842. 28613909

[ref8] Desplanque L.; Bucamp A.; Troadec D.; Patriarche G.; Wallart X. In-Plane InSb Nanowires Grown by Selective Area Molecular Beam Epitaxy on Semi-Insulating Substrate. Nanotechnology 2018, 29, 305705 10.1088/1361-6528/aac321. 29738312

[ref9] Güniat L.; Caroff P.; i Morral A. F. Vapor Phase Growth of Semiconductor Nanowires: Key Developments and Open Questions. Chem. Rev. 2019, 119, 8958–8971. 10.1021/acs.chemrev.8b00649. 30998006

[ref10] Aseev P.; Wang G.; Binci L.; Singh A.; Martí-Sánchez S.; Botifoll M.; Stek L. J.; Bordin A.; Watson J. D.; Boekhout F.; Abel D.; Gamble J.; van Hoogdalem K.; Arbiol J.; Kouwenhoven L. P.; de Lange G.; Caroff P. Ballistic InSb Nanowires and Networks via Metal-Sown Selective Area Growth. Nano Lett. 2019, 19, 9102–9111. 10.1021/acs.nanolett.9b04265. 31730748

[ref11] Tutuncuoglu G.; de la Mata M.; Deiana D.; Potts H.; Matteini F.; Arbiol J.; i Morral A. F. Towards Defect-Free 1-D GaAs/AlGaAs Heterostructures Based on GaAs Nanomembranes. Nanoscale 2015, 7, 19453–19460. 10.1039/C5NR04821D. 26416625

[ref12] Seidl J.; Gluschke J. G.; Yuan X.; Tan H. H.; Jagadish C.; Caroff P.; Micolich A. P. Postgrowth Shaping and Transport Anisotropy in Two-Dimensional InAs Nanofins. ACS Nano 2021, 15, 7226–7236. 10.1021/acsnano.1c00483. 33825436

[ref13] Shi Y.; Wang Z.; van Campenhout J.; Pantouvaki M.; Guo W.; Kunert B.; van Thourhout D. Optical Pumped InGaAs/GaAs Nano-Ridge Laser Epitaxially Grown on a Standard 300-Mm Si Wafer. Optica 2017, 4, 1468 10.1364/optica.4.001468.

[ref14] Friedl M.; Cerveny K.; Weigele P.; Tütüncüoglu G.; Martí-Sánchez S.; Huang C.; Patlatiuk T.; Potts H.; Sun Z.; Hill M. O.; Güniat L.; Kim W.; Zamani M.; Dubrovskii V. G.; Arbiol J.; Lauhon L. J.; Zumbühl D. M.; i Morral A. F. Template-Assisted Scalable Nanowire Networks. Nano Lett. 2018, 18, 2666–2671. 10.1021/acs.nanolett.8b00554. 29579392

[ref15] Vaitiekėnas S.; Whiticar A. M.; Deng M. T.; Krizek F.; Sestoft J. E.; Palmstrøm C. J.; Marti-Sanchez S.; Arbiol J.; Krogstrup P.; Casparis L.; Marcus C. M. Selective-Area-Grown Semiconductor-Superconductor Hybrids: A Basis for Topological Networks. Phys. Rev. Lett. 2018, 121, 147701 10.1103/PhysRevLett.121.147701. 30339420

[ref16] Lapierre R. R.; Robson M.; Azizur-Rahman K. M.; Kuyanov P. A Review of III-V Nanowire Infrared Photodetectors and Sensors. J. Phys. D: Appl. Phys. 2017, 50, 123001 10.1088/1361-6463/aa5ab3.

[ref17] Krizek F.; Sestoft J. E.; Aseev P.; Marti-Sanchez S.; Vaitiekenas S.; Casparis L.; Khan S. A.; Liu Y.; Stankevič T.; Whiticar A. M.; Fursina A.; Boekhout F.; Koops R.; Uccelli E.; Kouwenhoven L. P.; Marcus C. M.; Arbiol J.; Krogstrup P. Field Effect Enhancement in Buffered Quantum Nanowire Networks. Phys. Rev. Mater. 2018, 2, 093401 10.1103/PhysRevMaterials.2.093401.

[ref18] Friedl M.; Cerveny K.; Huang C.; Dede D.; Samani M.; Hill M. O.; Morgan N.; Kim W.; Güniat L.; Segura-Ruiz J.; Lauhon L. J.; Zumbühl D. M.; i Morral A. F. Remote Doping of Scalable Nanowire Branches. Nano Lett. 2020, 20, 3577–3584. 10.1021/acs.nanolett.0c00517. 32315191

[ref19] Albani M.; Ghisalberti L.; Bergamaschini R.; Friedl M.; Salvalaglio M.; Voigt A.; Montalenti F.; Tütüncüoglu G.; i Morral A.; Miglio L. Growth Kinetics and Morphological Analysis of Homoepitaxial GaAs Fins by Theory and Experiment. Phys. Rev. Mater. 2018, 2, 093404 10.1103/PhysRevMaterials.2.093404.

[ref20] Dede D.; Glas F.; Piazza V.; Morgan N.; Friedl M.; Güniat L.; Dayi E. N.; Balgarkashi A.; Dubrovskii V. G.; i Morral A. F. Selective Area Epitaxy of GaAs: The Unintuitive Role of Feature Size and Pitch. Nanotechnology 2022, 33, 485604 10.1088/1361-6528/ac88d9. 35952545

[ref21] Tausch F. W. Jr.; Lapierre A. G. III A Novel Crystal Growth Phenomenon: Single Crystal GaAs Overgrowth onto Silicon Dioxide. J. Electrochem. Soc. 1965, 112, 706–709. 10.1149/1.2423670.

[ref22] Pristovsek M.; Poser F.; Richter W. The Impact of the Surface on Step-Bunching and Diffusion of Ga on GaAs (001) in Metal-Organic Vapour Phase Epitaxy. Mater. Res. Express 2016, 3, 075902 10.1088/2053-1591/3/7/075902.

[ref23] Reichert W.; Cohen R. M. Lateral OMVPE Growth of GaAs on Patterned Substrates. J. Cryst. Growth 2000, 220, 364–378. 10.1016/S0022-0248(00)00829-0.

[ref24] Noborisaka J.; Motohisa J.; Fukui T. Catalyst-Free Growth of GaAs Nanowires by Selective-Area Metalorganic Vapor-Phase Epitaxy. Appl. Phys. Lett. 2005, 86, 213102 10.1063/1.1935038.

[ref25] Dubrovskii V. G. Theory of MOCVD Growth of III-V Nanowires on Patterned Substrates. Nanomaterials 2022, 12, 2632 10.3390/nano12152632. 35957064 PMC9370533

[ref26] Chi C. Y.; Chang C. C.; Hu S.; Yeh T. W.; Cronin S. B.; Dapkus P. D. Twin-Free GaAs Nanosheets by Selective Area Growth: Implications for Defect-Free Nanostructures. Nano Lett. 2013, 13, 2506–2515. 10.1021/nl400561j. 23634790

[ref27] Mangum J. S.; Theingi S.; Steiner M. A.; McMahon W. E.; Warren E. L. Development of High-Efficiency GaAs Solar Cells Grown on Nanopatterned GaAs Substrates. Cryst. Growth Des. 2021, 21, 5955–5960. 10.1021/acs.cgd.1c00835.

[ref28] Orme C.; Johnson M. D.; Sudijono J. L.; Leung K. T.; Orr B. G. Large Scale Surface Structure Formed during GaAs (001) Homoepitaxy. Appl. Phys. Lett. 1994, 64, 860–862. 10.1063/1.111004.

[ref29] Johnson M. D.; Orme C.; Hunt A. W.; Graff D.; Sudijono J.; Sander L. M.; Orr B. G. Stable and Unstable Growth in Molecular Beam Epitaxy. Phys. Rev. Lett. 1994, 72, 116–119. 10.1103/PhysRevLett.72.116. 10055580

[ref30] Bluhm H.; Schwarz U. D.; Herrmann F.; Paufler P. Study of the Influence of Native Oxide Layers on Atomic Force Microscopy Imaging of Semiconductor Surfaces. Appl. Phys. A: Solids Surf. 1994, 59, 23–27. 10.1007/BF00348415.

[ref31] Asai H. Anisotropic Lateral Growth in GaAs MOCVD Layers on (001) Substrates. J. Cryst. Growth 1987, 80, 425–433. 10.1016/0022-0248(87)90091-1.

[ref32] Dubrovskii V. G. Theory of Diffusion-Induced Selective Area Growth of III-V Nanostructures. Phys. Rev. Mater. 2023, 7, 026001 10.1103/PhysRevMaterials.7.026001.

[ref33] Sobanska M.; Zytkiewicz Z. R.; Ekielski M.; Klosek K.; Sokolovskii A. S.; Dubrovskii V. G. Surface Diffusion of Gallium as the Origin of Inhomogeneity in Selective Area Growth of GaN Nanowires on AlxOy Nucleation Stripes. Cryst. Growth Des. 2020, 20, 4770–4778. 10.1021/acs.cgd.0c00530.

[ref34] Dubrovskii V. G. Nucleation Theory and Growth of Nanostructures; Springer: Heidelberg – New York – Dordrecht – London, 2014.

[ref35] Cachaza M. E.; Christensen A. W.; Beznasyuk D.; Særkjær T.; Madsen M. H.; Tanta R.; Nagda G.; Schuwalow S.; Krogstrup P. Selective Area Growth Rates of III-V Nanowires. Phys. Rev. Mater. 2021, 5, 094601 10.1103/PhysRevMaterials.5.094601.

