# Supplementary material for: From Layer-by-Layer Growth to Nanoridge Formation: Selective Area Epitaxy of GaAs by MOVPE
Source: Cryst Growth Des. 2023 Jun 12;23(7):5083–92. doi: 10.1021/acs.cgd.3c00316 (PMC10326851; doi:10.1021/acs.cgd.3c00316)
Supplement: Supplementary file 1 — cg3c00316_si_001.pdf [file cg3c00316_si_001.pdf]

Supporting Information

From layer-by-layer growth to nanoridge formation: selective area epitaxy of GaAs by MOVPE

Nicholas Morgan[†], Vladimir G. Dubrovskii[‡], Ann-Kristin Stief[†], Didem Dede[†], Marie Sanglé-Ferrière[†], Alok Rudra[§], Valerio Piazza[†], Anna Fontcuberta i Morral[†,§]

[†] *Laboratory of Semiconductor Materials, Institute of Materials, EPFL, 1015 Lausanne, Switzerland*

[‡] *Faculty of Physics, St. Petersburg State University, Universitetskaya Embankment 13B, 199034 St. Petersburg, Russia*

[§] *Laboratory of Semiconductor Materials, Institute of Physics, EPFL, 1015 Lausanne, Switzerland*

$[\mathbf{010}]$ versus $[\mathbf{01\bar{1}}]$ Growth Directions

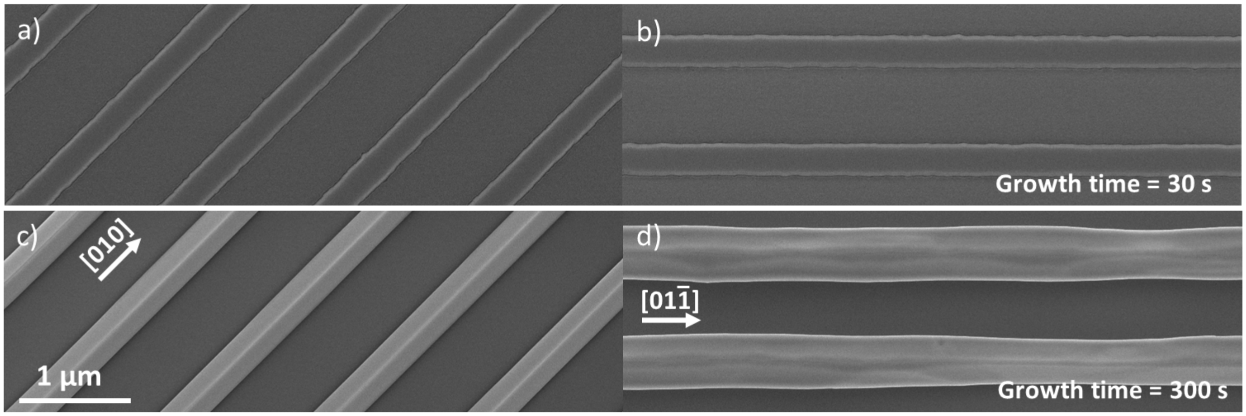

Figure S1. *Top view SEM images of GaAs nanoridges oriented along* $[010]$ *(a,c) and* $[01\bar{1}]$ *(b,d) directions, after 30 s (a,b) and 300 s (c,d) of growth.*

For short growth times, nanoridges along both $[010]$ and $[01\bar{1}]$ directions exhibit a flat top facet and angled side facets. For longer growth times, the $[01\bar{1}]$-oriented nanoridges become irregular with less well-defined side facets.

Atomic Steps After Annealing

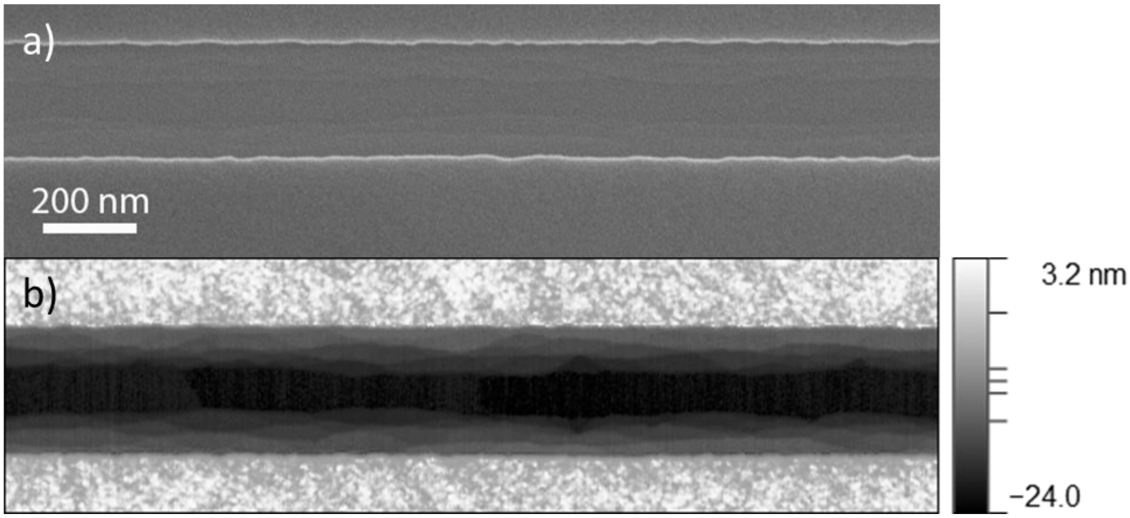

Figure S2. *a) Top view SEM image of an annealed trench with nominal pitch 1000 nm and nominal width 200 nm. b) AFM contour map of an annealed trench with nominal pitch 1000 nm and nominal width 240 nm, using adaptive non-linear coloring to highlight atomic steps. The scale bar is the same for both.*

After annealing, atomic steps are observed which run along the length of the trench. These steps are observed for all widths and pitches.

AFM Data Processing

Raw AFM data is processed first with Gwyddion. The procedure is as follows: drift correction, mean plane subtraction, leveling rows using intersections with 4 lines drawn top to bottom on the oxide mask, align rows, level data by fitting a plane through three points.

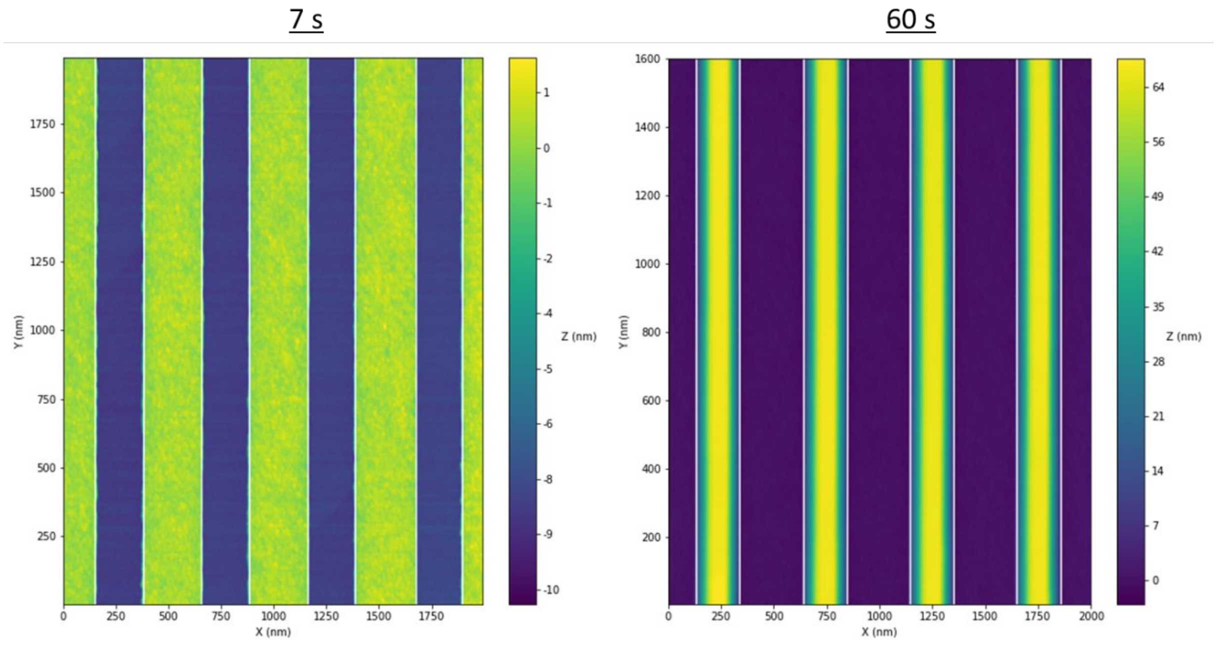

Figure S3. *AFM contour maps for array of trenches with nominal width 160 nm and pitch of 500 nm. The white lines are the fitted edge markers for measuring the width.*

Using edge detection, lines (shown in white) are fitted to mark the edge of the trench. These lines define the area which is considered for the calculation of the width of the structure. The maximum surface area is defined as this width multiplied by the length considered in the measurement. For nanostructures which still exhibit a flat (100) top facet, the measured height is taken as the height at the center point between these lines, averaged along the length of the nanoridge, with the mask level set to 0. For nanostructures which are fully formed (exhibiting only $\{101\}$ facets), the measured height is the maximum height of the nanoridge, averaged along its length.

In the model, H is taken as the height from the bottom of the annealed trench to the top of the nanostructure. Thus, the reported height H corresponds to the difference between the measured height of the grown nanostructure and the depth of the annealed trench with corresponding width and pitch. All measurements are taken from the center of the arrays, in order to avoid the influence of edge effects as described in Figure 4 of the main text.

Trench Depth Before Annealing

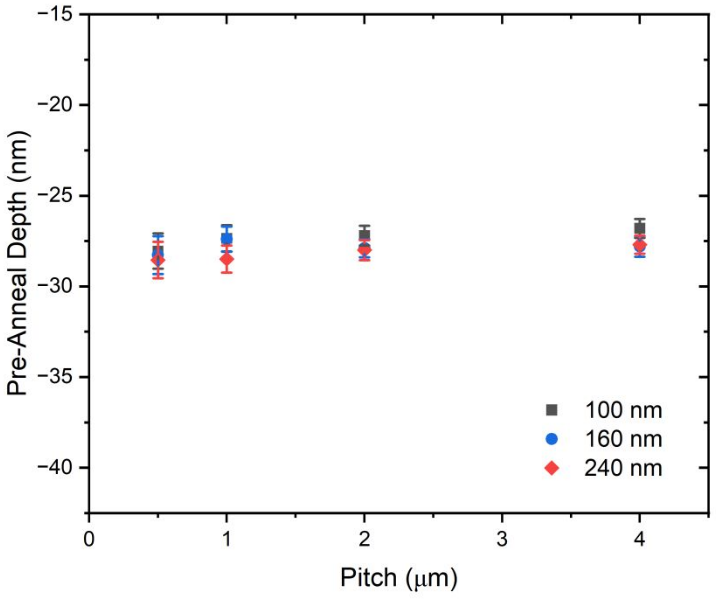

Figure S4. *Depth of as-prepared substrates, before annealing, as a function of pitch. The overall average depth is -28 nm.*

The depth of trenches from the as-prepared substrate was measured before annealing, exhibiting a very weak dependence on width and pitch. Any variation in depth is attributed to non-idealities of the reactive ion etching process.

Effect of Pitch on Height and Width

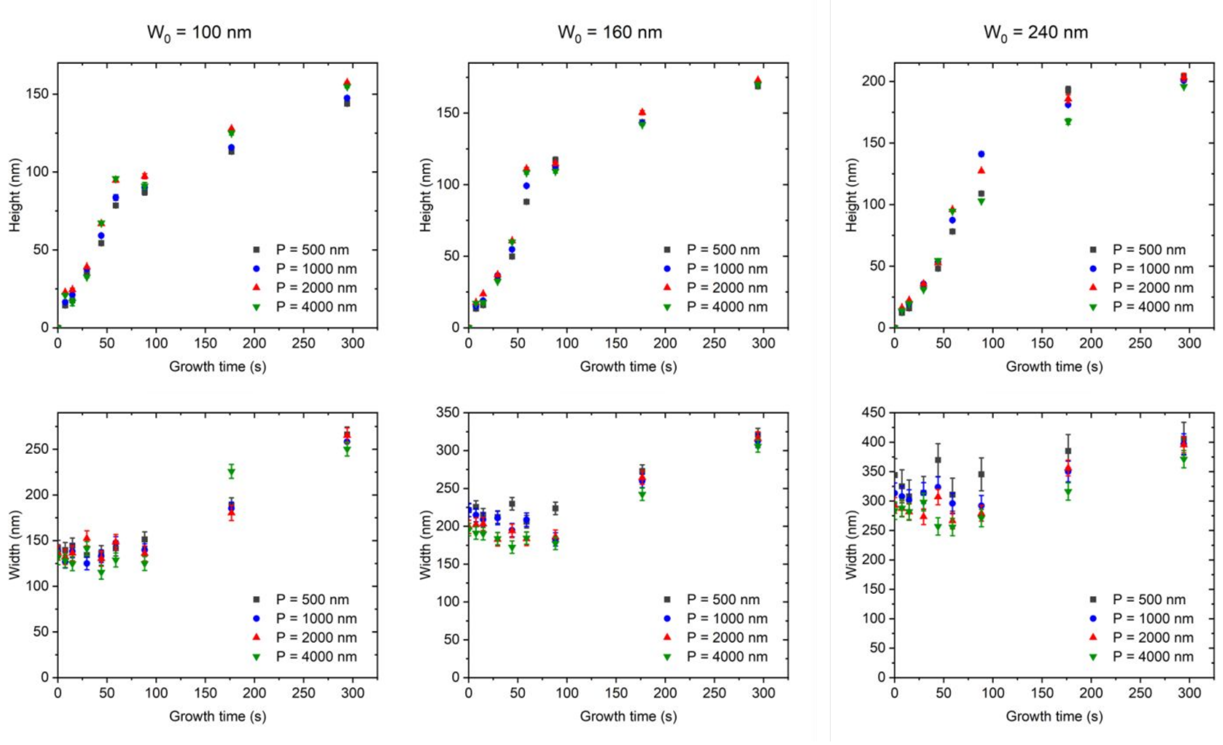

Figure S5. *Plots of measured height and width as a function of growth time for P = 500, 1000, 2000 and 4000 nm and for* W_0 *= 100, 160 and 240 nm.*

Height and width measurements were taken for pitches of 500, 1000, 2000 and 4000 nm and widths of 100, 160 and 240 nm. No clear correlation was found between pitch and height. There is a small correlation between pitch and width; smaller pitches yield larger widths. This is attributed to substrate processing effects, such as proximity effect during EBL, which leads to overexposure in denser patterns, causing a widening of the features. For this reason, modeling was done for the data set from one single pitch (P = 1000 nm), to avoid any confounding factors.
